# In Vitro Production and Exudation of 20-Hydroxymaytenin from *Gymnosporia heterophylla* (Eckl. and Zeyh.) Loes. Cell Culture

**DOI:** 10.3390/plants10081493

**Published:** 2021-07-21

**Authors:** Thanet Pitakbut, Michael Spiteller, Oliver Kayser

**Affiliations:** 1 Technical Biochemistry, Department of Biochemical and Chemical Engineering, TU Dortmund University, 44227 Dortmund, Germany; thanet.pitakbut@tu-dortmund.de; 2 Institute of Environmental Research (INFU), Department of Chemistry and Chemical Biology, TU Dortmund University, 44227 Dortmund, Germany; michael.spiteller@tu-dortmund.de

**Keywords:** plant cell culture, plant exudate, 20-hydroxymaytenin, *Gymnosporia heterophylla*

## Abstract

The metabolite 20-Hydroxymaytenin (20-HM) is a member of the quinone-methide pentacyclic triterpenoids (QMTs) group. This metabolite group is present only in Celastraceae plants, and it has shown various biological activities from antioxidant to anticancer properties. However, most QMTs metabolites including 20-HM cannot be synthesized in a laboratory. Therefore, we optimized a plant tissue culture protocol and examined the potential of *Gymnosporia heterophylla* (synonym. *Maytenus heterophylla*) to produce 20-HM in an in vitro experiment. For the first time, we reported the optimum callus induction medium with a high percentage success rate of 82% from the combination of 1 mg/L indole-3-butyric acid and 5 mg/L naphthalene acetic acid. Later, our cell suspension culture cultivated in the optimum medium provided approximately 0.35 mg/g fresh weight of 20-HM. This concentration is roughly 87.5 times higher than a concentration of 20-HM presenting in *Elaeodendron croceum* (Celastraceae) leaves. In addition, we also found that 20-HM presented in a cultivation medium, suggesting that *G. heterophylla* cells secreted 20-HM as an exudate in our experiment. Noticeably, 20-HM was missing when *Penicillium* cf. *olsonii* occurred in the medium. These findings hint at an antifungal property of 20-HM.

## 1. Introduction

The triterpenoid metabolite 20-Hydroxymaytenin (20-HM) belongs to a unique group known as quinone-methide pentacyclic triterpenoids or QMTs [1]. In previous reports, a wide range of biological activities was reported from QMTs metabolites, such as antioxidant [2,3], anti-inflammatory [4,5], antimicrobial [6,7], and antidiabetes [8]. In addition, QMTs metabolites also provided hepato- and cardioprotective effects [9,10]. Among all these biological activities, an anticancer property is the most promising one [11]. Even though no QMTs derivative has been brought to a clinical trial yet, QMTs metabolites, especially celastrol, are known as a hit compound for developing new anticancer agents [11,12,13]. Recently, Hernandes et al. (2020) reported that two bioactive QMTs metabolites from *Maytenus ilicifolia* (Celastraceae), namely maytenin and 22-hydroxymaytenin (22-HM), exhibited a promising antineoplastic effect against head and neck squamous cell carcinoma [14], the sixth most common cancer in the world [15,16]. Moreover, it was indicated that maytenin could effectively and specifically target cancer cells. However, maytenin demonstrated an inferior effect compared to cisplatin (the standard treatment), but it also showed less toxicity [14]. Lastly, Hernandes and his team also suggested that maytenin might prevent cancer cells from resisting cisplatin treatment by downregulating microRNAs such as miR-21-5p [14].

Despite the attractive biological activities of the QMTs metabolites, research and development have been left behind. One of the major limiting factors is that QMTs metabolites cannot be synthesized chemically due to their structural complexity [17,18,19]. A possible way to obtain QMTs metabolites is plant extraction. The problem is a very low accumulation of QMT in the plant. For example, Yelani et al. (2010) reported that 14 mg of 20-HM was isolated from 3.7 kg of *Elaeodendron croceum* (Celastraceae) leaves only [20]. It is approximately 0.004 mg/g dry weight of 20-HM presenting in *E. croceum* leaves. However, Coppde et al. (2014) reported a higher concentration of 22-HM at 0.060 mg/g dry weight in one-year-old *M. ilicifolia* roots. The concentration of 22-HM is roughly a quarter of a total concentration of QMTs metabolites presenting in one-year-old *M. ilicifolia* roots at 0.234 mg/g dry weight. Noticeably, plant tissue culture has been commonly used to overcome this obstacle [21,22,23]. Since there is no standard protocol for a cell suspension culture from *Gymnosporia heterophylla* (synonym. *Maytenus heterophylla*), we worked to establish one. We evaluated the possibility of using *G. heterophylla* cell suspension culture as an alternative source for the production of 20-HM. We optimized a callus induction medium using various combinations of indole-3-butyric acid (IBA) and naphthalene acetic acid (NAA) and optimized the phytohormone concentration. Finally, a growth curve and biosynthetic rate of 20-HM in *G. heterophylla* cells were determined. Following a suggestion from Pina’s study, we also analyzed a chemical profile of a cultivation medium to track 20-HM since Pina and co-authors reported that an in vitro root culture of *Cheiloclinium cognatum* could secrete 22-HM (a derivative of 20-HM) extracellularly [24].

In this study, plant calli (groups of dedifferentiated cells) were initiated from leaves of *G. heterophylla* [25]. Callus culture of *G. heterophylla* was optimized and provided a high percentage success rate of 82%. In addition, a five-fold increase of the biomass was obtained from a cell suspension culture after five-week cultivation. Biomass provided 0.35 mg/g of 20-HM, being approximately 87.5 times more highly concentrated than previously reported by Yelani et al. (2010) [20]. Our result showed that the leaf cells of *G. heterophylla* produced and secreted 20-HM as an exudate in our in vitro culture, similar to Pina’s report [24].

## 2. Results

### 2.1. Isolation, Identification, and Proposed Mass Fragmentation of 20-HM from G. heterophylla

**Figure 1:**
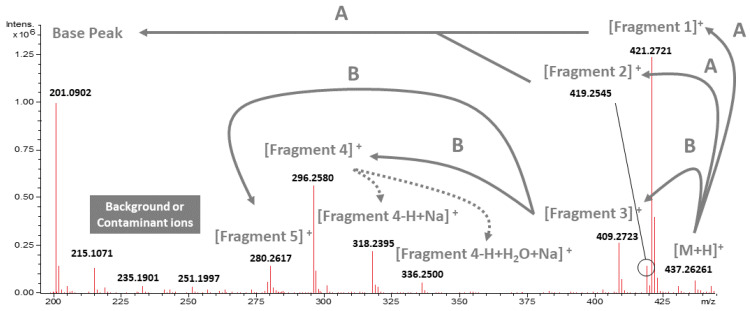
Observed ESI-MS spectrum of the obtained 20-HM and its proposed mass fragmentation. A and B indicate the mass fragmentation presenting in routes A and B from Figure 2 (below).

High purity of 20-HM is required as a standard reference for our chemical analysis, an essential part of this study. Since it is impossible to purchase any high purity of 20-HM from a company, we extracted, isolated, and identified 20-HM from *G. heterophylla.* As a result, 97% purity of 20-HM based on a high-performance liquid chromatography or HPLC analysis was obtained (see Supplementary Material, Figure S1). Later, ^13^C and ^1^H nuclear magnetic resonance or NMR spectroscopic data of the obtained 20-HM were compared to previous spectroscopic data reported by Gunatilka et al. (1989) presented in Table 1 [26]. As a result, a chemical structure of the obtained 20-HM from *G. heterophylla* was confirmed (see Supplementary Material, Figures S2 and S3). We proposed a mass fragmentation of 20-HM based on electrospray-ionization mass spectrometry or ESI-MS (Figure 1).

**Figure 2:**
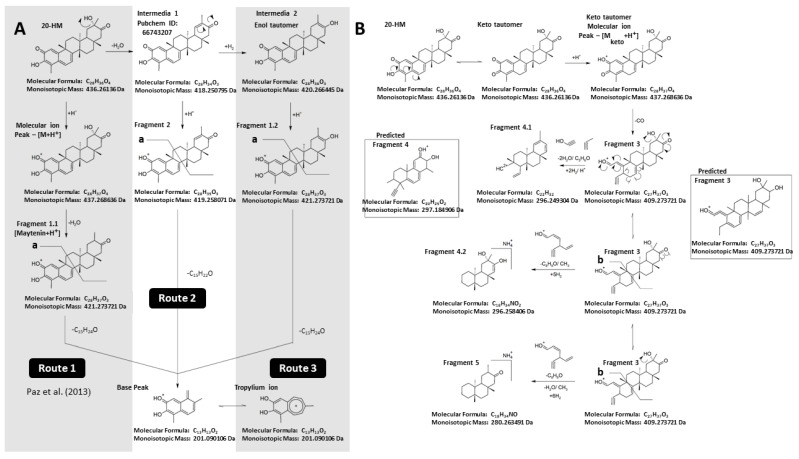
Proposed mass fragmentation scheme of 20-HM from a previous study and the PubChem database of 20-HM derivatives (**A**); proposed mass fragments by adopting CFM-ID program version 3.0 prediction (**B**). The predicted fragments of 20-HM from the CFM-ID program are shown in the boxes. (see Supplementary Material, Figure S4).

ESI-MS detected twelve ion peaks from 20-HM. Three of the peaks were assigned as background or contaminant peaks [27], and the remaining peaks (nine peaks) were proposed as a main fragmentation of 20-HM as presented in Figure 1; Figure 2 by combining a search through literature and chemical databases and a prediction from CFM-ID version 3.0 software [17,28,29]. Most of the proposed fragments were in a range of ±5 mDa from a theoretical mass calculated by ACD/Chemsketch (free version, 2015). Therefore, these fragments were accepted as an accuracy mass following a report of Watt et al. (2001) [30]. However, some ion peaks—namely a molecular ion peak, fragment 4.2, and fragment 4.2 with adduct ions—showed a difference greater than 5 mDa but less than 10 mDa. Therefore, they were still in a tolerated mass range reported by Zhang et al. (2018) [31]. A summary of the difference between theoretical and observed masses is provided in the Supplementary Material, Table S1.

### 2.2. Callus Induction Medium Optimization for Leaves of G. heterophylla

**Figure 3:**
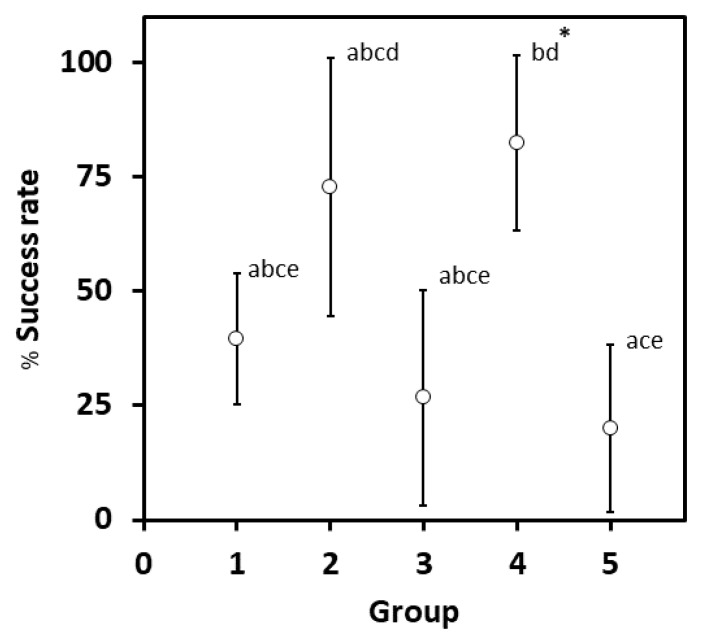
The percentage success rate of callus induction from *G. heterophylla* leaf cells in different treatment groups (MS medium supplemented with different levels of IBA and NAA as described in Table 1). The values present in the graph represent means with 95% confidence interval, mean ± 2SE as presented in Table 1. The same letters over the upper bar indicate the nonsignificant difference at *p*-value ≤ 0.05 among the groups according to the Tukey post-hoc test. * indicates the highest percentage success rate.

As introduced earlier, plant tissue culture techniques, such as callus and cell suspension cultures, have been commonly used to overcome a problem in a low production of the plant secondary metabolites from medicinal plants, and it has been shown to be realistic for Celastraece plants like *M. ilicifolia* and *C. cognatum* [14,24]. We optimized a cultivation medium by combining various concentrations of two phytohormones, such as indole-3-butyric acid (IBA) and naphthalene acetic acid (NAA), which provided a high percentage success rate in a callus formation from leaves of *G. heterophylla*. As presented in both Table 2 and Figure 3, the Murashige and Skoog (MS) medium supplemented with 1 mg/L IBA and 5 mg/L NAA was identified as the best medium.

Five different combinations of phytohormones in MS medium were selected and described in Table 2. The highest percentage success rate of 82.40% was found in group 4, MS medium supplemented with 1 mg/L IBA and 5 mg/L NAA. The second-highest percentage success rate of 72.70% was found in group 2, viz. MS medium supplemented with 3 mg/L IBA and 1 mg/L NAA. The remaining groups, such as groups 1, 3, and 5, showed a success rate below 50%. Based on the statistical analysis, as presented in Figure 3, there was no significant difference between the 2nd and 4th groups. However, only group 4 was significantly different from the other groups (group 1, 3, and 5). Therefore, the 4th group was defined as the optimum medium. Consequently, the MS recipe for group 4 was used in a cell suspension culture to evaluate the potential of *G. heterophylla* liquid cell culture to produce 20-HM. Usually, IBA and NAA were utilized to initiate hairy roots cultures [32,33,34], but here root formation was not observed (see Supplementary Material, Figure S5). However, past studies have shown success in the induction of callus formation from both IBA and NAA for some plants like *Ziziphora tenuior* (Lamiaceae), *Thymus persicus* (Lamiaceae), *Chirita swinglei* (Gesneriaceae), and *Brucea mollis* (Simaroubaceae) [35,36,37,38]. Therefore, IBA and NAA can be used for callus induction with favorable results.

### 2.3. Cell Suspension Culture of G. heterophylla and Evaluation of the Presence of 20-HM in Both Plant Cells and Cultivation Medium by HPLC-ESI-MS Analysis

**Figure 4:**
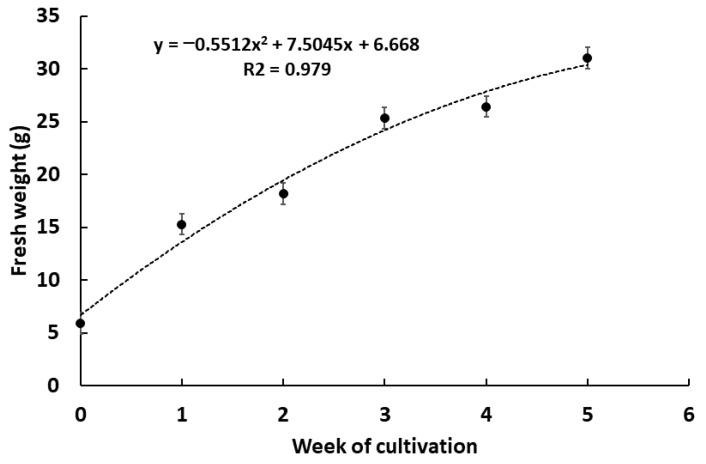
Growth curve of *G. heterophylla* cell suspension culture in the optimized cultivation medium. The fresh weight of the cells is monitored and presents in the mean ± SD from three replicates (*n* = 3). The non-linear regression (a quadratic model) is fitted with the growth curve as shown in the dotted line. The equation and R^2^ of the regression are also provided.

After the optimum medium was determined, a growth curve of a cell suspension culture from *G. heterophylla* was monitored for five weeks, and the presence of 20-HM in plant cells and cultivation medium was evaluated at the end of the experiment. The biomass of *G. heterophylla* increased approximately five-fold by the end of the study, from 5.94 ± 0.75 g to 31.06 ± 3.49 g (Figure 4). The growth curve was fitted with a quadratic regression model where R^2^ = 0.979. Post-cultivation, the *G. heterophylla* cells and the liquid medium were separated by filtration and the cultivation medium was partitioned with ethyl acetate (EtOAC) and named a medium extract. Harvested cells were extracted with dichloromethane and called a cell extract. The HPLC-ESI-MS analysis of both cell and medium extracts was evaluated to identify 20-HM.

**Figure 5:**
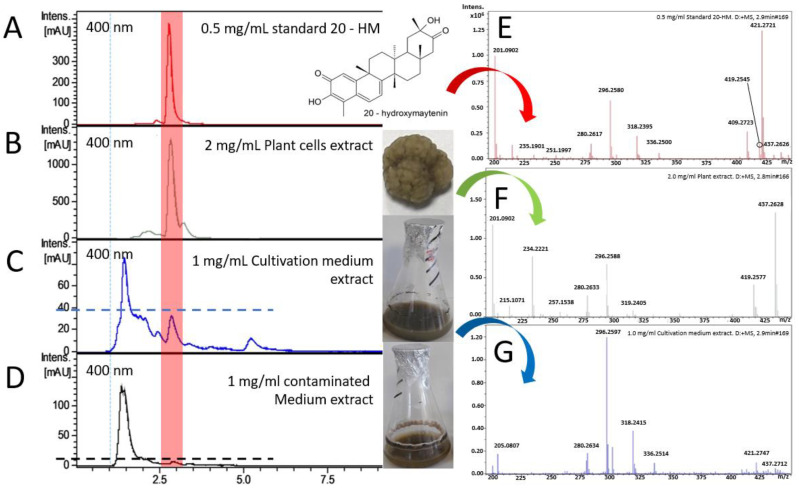
HPLC chromatograms and MS spectra of 0.5 mg/mL the standard 20-HM (**A**,**E**); 2 mg/mL *G. heterophylla* cells extract (**B**,**F**); 1 mg/mL cultivation medium extract (**C**,**G**); and 1 mg/mL infected medium (**D**). The highlighted red box indicates similar Rt values (2.8 to 2.9 min) between samples and the standard 20-HM.

Based on HPLC chromatograms (Figure 5A–C), 20-HM was detected in both cell and medium extracts at a retention time (Rt) of 2.8 and 2.9 min, matching with a standard reference. Moreover, the ESI-MS spectra of both extracts were highly similar to the reference. Therefore, we believe that 20-HM was presented in both plant and medium extracts. Trimpin et al. (2019) reported that the ESI-MS technique is sensitive, and components in a test sample (or a matrix) are essential because they can affect both the magnitude of an ion peak’s abandon (peak’ height) and the absence of some ion peaks in the MS spectra [39]. Noticeably, both plant and medium extracts are in a complex matrix (a mixture of compounds), unlike the reference that is in a more straightforward matrix (a pure compound). Therefore, some minor differences in the MS spectra between both extracts and the reference were expected. The concentration of 20-HM presenting in the cultivation medium was much lower than in the cell extract. However, this finding was in line with a previous study of *C. cognatum* by Pina et al. (2017) [24] and confirmed our hypothesis that *G. heterophylla* leaf cells were able to secret 20-HM like *C. cognatum* root cells.

Furthermore, Pina and co-authors suggested that an exudate from their study may have ecological functions to promote plant benefits, and one of the possible functions is repelling microorganisms [24]. Following Pina’s suggestion, we decided to evaluate the presence of 20-HM in a contaminated culture medium. As shown in Figure 5D, nearly none of 20-HM was found in the contaminated medium. Later, a fungus that caused this contamination was isolated and identified as *Penicillium* cf. *olsonii* by a PCR assay using the internal transcribed spacer (ITS) region as a marker (GeneBank accession number: MZ297876.1). Details regarding the identification of this fungus are provided in the Supplementary Material, Figure S6. In addition, missing 20-HM in the contaminated medium by *P.* cf. *olsonii* (Figure 5D) but not in the normal medium (Figure 5C) hinted at a biological activity of 20-HM as a biocontrol agent as proposed by Pina et al. (2017) [24]. However, more investigations are required to determine the relationship between *G. heterophylla,* 20-HM, and *P.* cf. *olsonii*. The relationship between plant and *P. olsonii* remains unclear, and more data are needed. For example, Latz et al. (2020) reported a mutualistic effect between *P. olsonii* and *Triticum aestivum* [40], but Punja et al. (2019) presented a pathogenic effect of *P. olsonii* in *Cannabis sativa* [41]. Based on the work of Kogel et al. (2006), a fungus may transit from symbiosis to pathogenesis or endophytism to parasitism if an imbalance condition allows the fungus to take over its plant host [42].

Finally, we isolated 20-HM from our *G. heterophylla* cell suspension culture. As a result, 17 mg of 20-HM was isolated from 20 g fresh weight of *G. heterophylla* cells. Based on the obtained amount of 20-HM, we estimated that our *G. heterophylla* cells produced 20-HM approximately 0.35 mg/g fresh weight, indicating a significantly higher concentration of 20-HM from nature as reported by Yelani et al. (2010) [20]. In addition, our *G. heterophylla* cells also provided a greater concentration of 20-HM than a total concentration of QMTs metabolites presenting in one-year-old *M. ilicifolia* roots, a close species of *G. heterophylla* (synonym: *M. heterophylla*); approximately 1.49 times higher concentration than a report from Coppde et al. (2014) [21]. In his report, the total concentration of the QMTs metabolites was presented at 0.234 mg/g dry weight. Therefore, based on the concentration of 20-HM and the total concentration of QMTs metabolites in nature, it is clear that our in vitro *G. heterophylla* cell suspension culture can produce a higher concentration of 20-HM and be considered as an alternative source for 20-HM production.

### 2.4. Partial Sequences and Gene Expression of the Hypothetical FRS Gene from G. heterophylla

In previous studies, Souza-Moreira et al. (2016) and Alves et al. (2018) reported that the FRS gene plays a vital role in the biosynthesis of QMTs metabolites by encoding an essential enzyme producing a precursor [43,44]. However, the FRS gene from *G. heterophylla* is not yet reported. Until now, there are only nine deposited FRS sequences in the NCBI database, including one synthetic gene, five genes from *M. ilicifolia*, one gene from *Populus davidiana* (Salicaceae), another gene from *Kalanchoe daigremontiana* (Crassulaceae), and the last and latest gene from *Quercus suber* (Fagaceae). Therefore, we decided to amplify a part of the FRS gene from *G. heterophylla* using one of the reported FRS genes from *M. ilicifolia*, a close species, as a template (NCBI accession number: MG677133.1).

**Figure 6:**
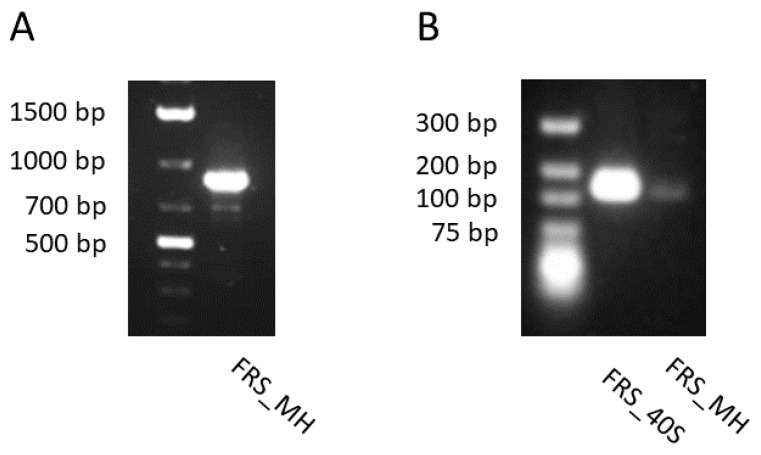
Amplified PCR product of the hypothetical FRS gene from the genomic DNA, spanning around 800 bp (**A**); and complementary DNA, spanning about 160 bp of *G. heterophylla* compares to 40S ribosomal protein as the reference gene, spanning around 160 bp (**B**).

**Figure 7:**
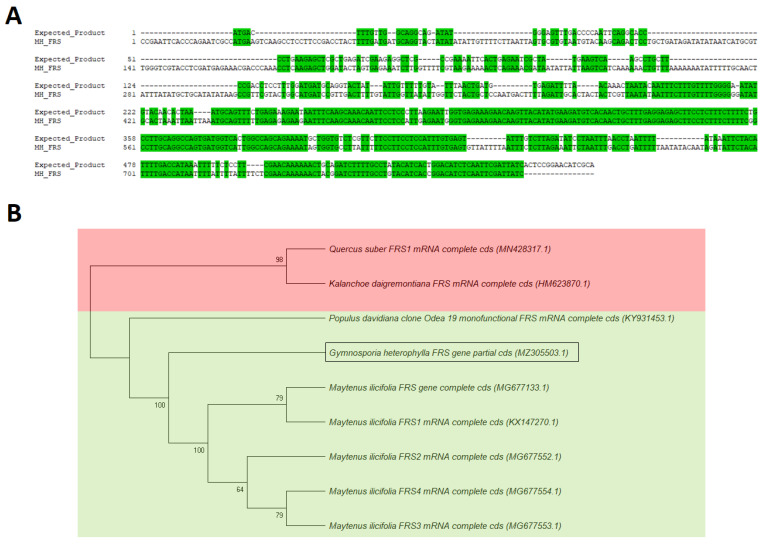
Pairwise alignment between the template PCR product of the FRS gene from *M. ilicifolia* and isolated PCR product of the FRS gene from *G. heterophylla*. Green highlights indicate the conserved bases (**A**); phylogenetic analysis of the obtained friedelin synthase (FRS) gene from *G. heterophylla,* and deposited FRS gene sequences in the NCBI database (**B**). This tree is deposited on the TreeBASE database (TreeBASE tree ID: tr131454). Multiple alignments are performed using the Muscle tool, and the phylogenic tree was constructed using the maximum parsimony (MP) method from MEGA-X software (Version 10.0.4). The bootstrap values are shown on the branch based on 1000 pseudoreplicates. The MP tree is obtained by applying the min-mini heuristic algorithm with a searching factor of 1. The green box indicates a shared cluster among the relevant plant species, while the red box indicates a non-related cluster of the FRS genes. A box indicates an obtained FRS gene from *G. heterophylla* (NCBI accession number: MZ305503.1).

As presented in Figure 6A, a part of the FRS gene from *G. heterophylla* was amplified, spanning around 800 bp from a genomic material. After that, the FRS gene was also amplified from a transcriptomic material of *G. heterophylla*, spanning around 160 bp (Figure 6B). In parallel, the amplified PCR product from a genomic DNA of *G. heterophylla* was sequenced, and an obtained FRS gene sequence was aligned with the template from *M. ilicifolia*. As shown in Figure 7A, the obtained FRS gene sequences were approximately 60% conserved with the template. Later, a phylogenetic tree was constructed to observe a correlation between the obtained partial FRS gene from *G. heterophylla* and the other eight deposited FRS genes from the NCBI database, except the synthetic FRS gene, which was excluded (Figure 7B). Alves et al. (2018) reported that FRS genes from *M. ilicifolia* and *P. davidiana* shared the same cluster, while the FRS gene from *K. daigremontiana* was not included [44]. Our phylogenetic analysis provided the same result as Alves et al. (2018) and indicated that our partial FRS gene from *G. heterophylla* also shared the same cluster with *M. ilicifolia*, the close species and our template, and *P. davidiana* (Figure 7B, green box). Furthermore, this phylogenetic analysis suggested a high correlation between the FRS genes from *K. daigremontiana* and *Q. suber*, the latest FRS gene reported by Busta et al. (2020), as shown in Figure 7B (red box) [45]. In conclusion, we report partial sequences of the hypothetical FRS gene from *G. heterophylla* based on a PCR assay and phylogenic analysis.

## 3. Discussion

Recently, various biological activities from QMTs metabolites have been reported, namely antioxidant [2,3], antimicrobial [6,7], and anticancer properties [11]. However, our understanding of QMTs metabolites’ bioactivities is still far from complete, especially regarding the activities of rare QMTs metabolites like 20-HM. Based on a previous report by Yaleni (2010), only 0.004 mg/g dried weight of 20-HM was presented in leaves of *E. croceum* [20]. We used *G. heterophylla* as a model and optimized a plant tissue culture protocol to increase the production of 20-HM. Our optimized protocol provided a high percentage success rate of 82% in a callus induction step and increased a significant concentration of 20-HM from nature by approximately 87.5 times. A low detectable concentration of 20-HM was found in the cultivation medium. This finding indicates that 20-HM was excreted from *G. heterophylla* cell culture and has shown a possibility for a non-invasive bioprocess to obtain 20-HM from *G. heterophylla* cell suspension culture in the future. In parallel, the low concentration of 20-HM in the medium hinted at a biological function of 20-HM as a biocontrol agent suggested by Pina et al. (2017) [24].

Based on previous reports by Luo et al. (2005) and Mahlo and Eloff (2014), the studies indicated that 20-HM’s derivatives (prismertin and celastrol) and other pentacyclic triterpenoids, such as urosolic acid, have antifungal properties against phytopathogenic fungi, namely *Blumeria graminis, P. expansum, P. janthinellum,* and *P digitatum* [46,47]. A very recent study from Nikolova et al. (2021) also reported that urosolic acid was secreted as an exudate from an aerial part of *Origanum dictamnus* and *O. vulgarei* [48]. We suggest that 20-HM has a potential antifungal property and speculate that a possible role such antifungal activity of 20-HM is to block the active site of 14α-demethylase (14DM). We performed the first molecular docking studies as proof of our concept of 14DM based inhibition (see Supplementary Material, Figure S7). The enzyme 14DM is responsible for cell wall formation and conserved among the eukaryotic cells, including *Penicillium* species. Further, 14DM inhibition results in a stop of cell wall formation and inhibits fungal cell growth [49,50]. Our docking result showed that 20-HM may block the active site of 14DM in the same manner as fluquinconazole (an agricultural antifungal agent and potent 14DM inhibitor). All docking results are presented in the Supplementary Material, Figure S8. However, an in vitro antifungal assay was not carried out due to a lack of sufficient amounts of 20-HM.

We also report the partial sequences of the hypothetical FRS gene and its gene expression from *G. heterophylla* for the first time. We could only amplify an 800 bp fragment, but this fragment was long enough to identify as the hypothetical FRS gene based on the Blastn and phylogenic analyses [51,52]. Since the current information of the FRS gene is very limited (only nine FRS genes from four plant species were reported), we believe that our report can provide more diversity in FRS sequences.

## 4. Materials and Methods

### 4.1. Plant Materials

*G. heterophylla* was the plant material that was used in this study. The plant was collected from South Africa (latitude 33°27′ 32.29′ S and longitude 21°25′ 41.21′ E) and identified by Mr. Ulrich Feiter, Parceval, Wellington, South Africa. The plant was cultivated at Technical Biochemistry, Department of Biochemical and Chemical Engineering, TU Dortmund University, Dortmund, Germany (voucher number: GH-CHEM-2017). Two DNA barcodes, namely rcbL and matK genes, were amplified and sequenced for a DNA fingerprint record. Phylogenic analysis of the obtained sequences and the best hits from the NCBI Blastn search (https://blast.ncbi.nlm.nih.gov/Blast.cgi, accessed on 15 April 2021) were constructed. The obtained gene fragments were deposited in the NCBI database (NCBI accession numbers: MZ305504.1 and MZ305505.1), and the phylogenetic trees were deposited on the TreeBASE database (TreeBASE tree ID: Tr131454 and Tr131462). All of the phylogenic trees can be found in the Supplementary Material, Figures S9 and S10.

### 4.2. Callus Induction and Cell Suspension Culture of G. heterophylla

A callus induction experiment was modified from previous studies [17,18,21,24], and the details of the experiment are described. *G. heterophylla* leaves were collected and immersed in 70% ethanol for 1 min before subjected to 1% active chlorine of sodium hypochlorite with a few drops of Tween 20 for 10 min. After that, the samples were washed three times in sterile water and dried. The sterilized leaves were cut into small pieces approximately 1–2 cm, known as explants, and placed in MS agar medium (4.4 g/L MS medium with 30 g/L sucrose at pH 5.8 supplemented with 0.5 mg/L 6-benzylaminopurin in 8 g/L agar). To optimize the medium, various combinations of IBA and NAA were assessed—such as 5 mg/L IBA, 3 mg/L IBA: 1 mg/L NAA, 2 mg/L IBA: 2 mg/L NAA, 1 mg/L IBA: 5 mg/L NAA, and 5 mg/L NAA—and named as groups 1–5 respectively. These explants were cultivated at 25 °C under a 16 h light/8 h dark photoperiod. Callus cultures were cultivated for eight weeks, and every four weeks, the cultivation mediums were refreshed. At the end of week eight, a percentage success rate in a callus formation of each group was evaluated.

For the cell suspension culture, approximately 6 g of plant calli from the optimized callus medium was cut into small pieces and transferred to 250 mL Erlenmeyer flasks filled with 50 mL of the same liquid MS medium used earlier. These liquid mediums were maintained on an orbital shaker at 150 rpm. The MS medium was refreshed every week and cultivated for five weeks. At the end of week five, 20 g of plant cells and the cultivation medium were collected. These two samples were extracted and evaluated for the presence of 20-HM.

### 4.3. HLPC-ESI-MS Analysis

We used an Agilent 1290 HPLC system (Agilent, Waldbronn, Germany) coupled to a high–resolution LTQ-Orbitrap XL Mass spectrometer (Thermo Fisher Scientific, Waltham, MA, USA) equipped with a HESI-II ion source (Thermo Fisher Scientific, Waltham, MA, USA) for our chemical analysis. Acquisition by the DAD detector was performed at 400 nm and by the mass spectrometer, in positive mode from 90 to 900 m/z. The source parameters were established similar to previous publications [53,54,55]: 4 bar for nebulizing gas pressure, 12 L/min for a dry gas (N2) flow, 220 °C for the desolvation gas (N2) temperature, and 4.5 kV for a capillary voltage.

In parallel, we modified the HPLC condition from previous studies [17,21]. In brief, we injected 5 µL of 1 to 2 mg/mL medium and plant extracts into the Nucleodur RP 18 EC column (2 × 100 mm, i.d.; 2.7 µm). For UV detection, we used the detection wavelength at 400 nm. Moreover, we mixed 85% methanol with 0.1% formic acid and 15% water with 0.1% formic acid and used them as a mobile phase. Finally, we operated the HPLC analysis for 20 min at the flow rate of 0.2 mL/min under isocratic elution.

### 4.4. Isolation, Purification, and Structural Determination of 20—HM

Isolation and purification protocols of 20-HM follow a previous work from the authors [56]. The protocols are described here. In short, 20 g of fresh *G. heterophylla* cells were homogenized in liquid nitrogen. This homogenized sample was extracted with CH_2_Cl_2_ three times. After that, all extracts were combined and concentrated in a rotary evaporator. This CH_2_Cl_2_ extract was resuspended in a mixture of 9:1 methanol:water until it reached a 5 mg/mL concentration. After that, the resuspended solution was loaded into the preconditioned SPE column, C-18 column 500 mg/6 mL from Macherey Nagel (Düren, Germany), with the same solvent system as mentioned earlier. For the elution step, a mixture of 8:2 methanol:water was eluted until the yellow band came out. This yellow fraction was once again combined and concentrated before loading on a preparative thin-layer chromatography (prep TLC). This prep TLC was developed five times with a mixture of 8:2 hexane:chloroform. The yellow band was collected from the prep TLC and extracted by chloroform. Finally, this yellow substance was purified by Sephadex LH-20 column before subjected to chemical structure determination through spectroscopy. In the end, 7 mg of purified substance were obtained, and its spectroscopic data were compared to previous reports [17,18,21,24,26].

### 4.5. Isolation and Identification of the Parasitic Fungus

An isolation protocol for the parasitic fungus was modified from previous studies, and the ribosomal nuclear internal transcribed spacer (ITS) region was used to identify the species of this fungus [57,58,59]. The fungus was collected from the cultivation flask and placed on a standard potato dextrose agar (PDA). After 14 days of cultivation, the genomic DNA of this parasitic fungus was extracted by using the NucleoSpin Microbial DNA mini kit from Macherey Nagel (Düren, Germany). This extraction was performed strictly following the guidelines from the manufacturer.

Amplification of the fungal ITS region was made by using the polymerase chain (PCR) reaction. For the PCR amplification, 0.5 µL of the ITS1-forward primer, TCCGTAGGTGAACCTGCGG, and 0.5 µL of the ITS2-reverse primer, GCTGCGTTCTTCATCG-ATGC, was added into the PCR tube following 2 µL of the fungal DNA template and the other 2 µL of the sterile water. Finally, 45 µL of the Red Taq polymerase Master Mix (1.1×) from VWR was added and mixed thoroughly. The PCR cycling comprised of 1 cycle of an initial denaturation (95 °C, 2 min), 35 cycles of denaturation (95 °C, 30 s), annealing (57 °C, 40 s), and elongation (72 °C, 1 min). Finally, the final elongation step was performed at 72 °C for 5 min. The PCR product from above was evaluated by using agarose gel electrophoresis. The expected band at around 600 bases was extracted and purified using Macherey Nagel Gel and PCR Clean-up (Düren, Germany). Consequently, the nucleotide sequences of the ITS as a DNA barcode marker were obtained from Microsynth/Seqlab (Goettingen, Germany). Eventually, the obtained sequences were deposited in the NCBI database (NCBI accession number: MZ297876.1). Phylogenic analysis of the obtained ITS region and the best hits from the NCBI Blastn search (https://blast.ncbi.nlm.nih.gov/Blast.cgi, accessed on 1 May 2021) was performed (see Supplementary Material, Figure S6). The phylogenic tree was deposited in the TreeBASE database (TreeBASE tree ID: Tr131453).

### 4.6. Primer Design and PCR Amplification of Partial Sequences of the Hypothetical FRS Gene from G. heterophylla

For the plant genomic DNA, fresh leaves of *G. heterophylla* were collected and immediately submerged into liquid nitrogen for grinding. The genomic DNA was extracted from the homogenized sample using the Macherey Nagel Nucleospin Plant II (Düren, Germany). The primer pair for the FRS gene amplification from *G. heterophylla* was designed based on a previous report of the complete FRS gene from *M. ilicifolia* [43,44]. Primer3web (http://primer3.ut.ee/, accessed on 25 February 2021) was used to design these primers. The forward and reverse primers were named FRS-MH-F (FRS-MH-F: ATGACTTTGTTGGCAGGCAG) and FRS-MH-R (FRS-MH-R: TGCGATGTTCCGGAGTGATA), respectively. The standard PCR reaction was performed as described in the previous experiment. Only an annealing temperature was adjusted to 65 °C, which is a suitable temperature for these primers. Finally, the amplified band at around 800 bases was extracted and purified using Macherey Nagel Gel and PCR Clean-up (Düren, Germany). Consequently, the nucleotide sequence was sent to Microsynth/Seqlab (Goettingen, Germany), and the obtained sequence was deposited in the NCBI database (NCBI accession number: MZ305503.1). Phylogenic analysis of the obtained FRS gene and the best hits from the NCBI Blastn search (https://blast.ncbi.nlm.nih.gov/Blast.cgi, accessed on 4 May 2021) was constructed. The phylogenic tree was deposited in the TreeBASE database (TreeBASE tree ID: Tr131441)

In parallel, the Nucleospin Plant RNA kit from Macherey Nagel (Düren, Germany) was used to extract the total RNA from the leaves of *G. heterophylla*. Before performing the PCR reaction, complementary DNA was synthesized using Luna Universal qPCR Master Mix (New England Biolabs, Frankfurt, Germany). For the PCR reaction, the same primers using in the previous experiment were also used here, following the same procedure. However, the annealing temperature was changed from 65 to 70 °C. For the reference gene, 40S ribosomal protein was selected as suggested in a previous study [43]. Finally, the PCR products were checked by using agarose gel electrophoresis.

### 4.7. DNA Barcoding Identification of G. heterophylla

Two DNA barcoding markers (rcbL and matK genes) that had been suggested from a previous study were used here [60]. A standard PCR reaction that was used earlier was also applied here. However, the annealing temperature was adjusted to 60 °C, which was suitable for primers of both rcbL and matK genes. The primer pair for rcbL gene was rbcL-F: ATGTCACCACAAACAGAGACTAAAGC as a forward primer and rbcL-R: GTAAAATCAAGTC-CACCRCG as a reverse primer. In comparison, the primer pair for matK gene was MatK-F: CGTACAGTACTTTTGTGTTTACGAG as a forward primer and MatK-R: ACCCAGTCCATCTGGAAATCTTGGTTC as a reverse primer. The expected PCR products of rcbL and matK genes were 600 and 800 bases, respectively. Finally, the amplified PCR products were isolated and sent to the same company for sequencing. The obtained sequences were was also deposited in the NCBI database (NCBI accession number: MZ305504.1 and MZ305505.1). Phylogenic analysis of the obtained rcbL and matK genes and the best hits from the NCBI Blastn search (https://blast.ncbi.nlm.-nih.gov/Blast.cgi, accessed on 15 April 2021) was done. The phylogenic tree was deposited in the TreeBASE database (TreeBASE tree ID: Tr131445 and Tr131462).

### 4.8. Data Availability

All gene sequences in this study were deposited in the NCBI database (https://www.ncbi.nlm.nih.gov/, accessed on 2 June 2021), such as ITS sequences of the isolated *P.* cf. *olsonii* from our culture (NCBI accession number: MZ297876.1), the FRS gene from our *G. heterophylla* (NCBI accession number: MZ305503.1), and two DNA barcode genes from *G. heterophylla* (NCBI accession number: MZ305504.1 and MZ305505.1). In addition, all phylogenic trees were deposited in the TreeBASE database with a study ID: S28331 (http://purl.org/phylo/treebase/phylows/study/TB2:S28331?x-access-ode=70da4e3f3a18e5e0392ed97bbc45f198&format=html, accessed on 2 June 2021).

### 4.9. Bioinformatic and Statistic Softwares

Clone manager version 9 professional edition (Scientific & Educational Software, USA) was used to visualize the nucleotide sequences and the phylogenic analysis was constructed by using the MEGA-X program (version 10.0.4) [61]. Next, mesquite software (version 3.61) was utilized to prepare each phylogenic file before a TreeBASE submission [62]. Finally, the Jamovi program (version 1.2.27) was used for the statistical analysis [63].

## Figures and Tables

**Table 1:** Comparison of ^13^C and ^1^H-NMR spectroscopic data between a previous report and a self-isolation of 20-HM from this study.

Position	13C—NMR		Position	1H—NMR					
	Reference *	Isolated		Reference *			Isolated		
	δ (ppm)	δ (ppm)		δ (ppm)	Multiplicity	*J* value (Hz)	δ	Multiplicity	*J* Value (Hz)
1	119.8	119.8	1	6.53	d	1.0	6.55	d	1.0
2	178.4	178.4	2						
3	146.2	146.1	3	6.97	s		6.69	s	
4	117.1	117.1	4						
5	127.9	127.7	5						
6	133.3	133.6	6	7.01	dd	7.0, 1,0	7.03	dd	7, 1
7	118.3	118.2	7	6.36	d	7.0	6.37	d	7.0
8	168.7	168.7	8						
9	42.9	42.7	9						
10	164.2	164.7	10						
11	33.2	32	11α	1.95	td	14.0, 7.0	1.95	m	
			11β	2.21	ddd	14.0, 5.0, 3.0	2.26	ddd	14.0, 5.0, 3.0
12	29.9	29.9	12α	1.76	ddd	14.0, 7.0, 3.0	1.76	m	
			12β	1.82	td	14.0, 5.0	1,79	m	
13	40	40.6	13						
14	44.2	44.6	14						
15	29.4	29.5	15α	1.84	ddd	12.5, 10.5, 4.5	1.83	m	
			15β	1.73	m		1.74	m	
16	35.7	34	16α	1.65	ddd	13.5, 4.5, 2.5	1.67	m	
			16β	1.91	ddd	13.5, 10.5, 5.5	1.86	m	
17	35.9	35.5	17						
18	43.3	43.5	18	1.93	dd	9.5	1.88	m	
19	36.9	38.2	19α	2.28	dd	15.0, 9.0	2.21	dd	15.0, 9.0
			19β	2.2	dd	15.0, 5.0	2.19	dd	15.0, 7.0
20	73.7	- ^¥^	20	3.24	s		3.5	d	5.0
21	214.9	213.6	21						
22	50.5	50.9	22α	2.99	d	14.0	2.93	d	14.0
			22β	1.95	d	14.0	1.99	m	
23	10.3	10.3	23	2.22	s		2.23	s	
24	-	-	24						
25	38.5	39	25	1.48	s		1.51	s	
26	23.3	21.5	26	1.36	s		1.35	s	
27	19.4	19.7	27	0.89	s		0.87	s	
28	33.2	32	28	1.13	s		1.26	s	
29	-	-	29	-			-	-	
30	29	28.5	30	1.36	s		1.35	s	

* indicates the spectroscopic data from a previous report [26]. ^¥^ indicates the quaternary carbon atom. s = singlet. d = doublet. dd = doublet of doublets. ddd = doublet of doublets of doublets. m = multiplet.

**Table 2:** Descriptive analysis of each treatment group and its percentage success rate in the callus formation.

Group	Description	Mean	SD	SE
1	5 mg/L IBA + 0 mg/L NAA	39.60	49.40	7.13
2	3 mg/L IBA + 1 mg/L NAA	72.70	46.70	14.08
3	2 mg/L IBA + 2 mg/L NAA	26.70	45.80	11.82
4	1 mg/L IBA + 5 mg/L NAA	82.40	39.30	9.53
5	0 mg/L IBA + 5 mg/L NAA	20.00	41.00	9.18

SD = standard deviation. SE = standard error of mean.

## Data Availability

Data reported are available in the Supplementary Materials.

## Supplementary Materials

The following are available online at https://www.mdpi.com/article/10.3390/plants10081493/s1, Figure S1: HPLC chromatogram of isolated 20-HM, Figure S2: ^1^H-NMR of 20-HM, Figure S3: ^13^C-NMR of 20-HM, Figure S4: Proposed MS fragmentation of fragment 3 and fragment 4.1, Figure S5: Callus and cell suspension cultures of *G. heterophylla*, Figure S6: Phylogenic tree of the ITS region of the isolated *P.* cf. *olsonii* from *G. heterophylla* cell culture, Figure S7: Docking poses of fluquinconazole and 20-HM, and their average binding energy (kcal/mol), Figure S8: 2D and 3D molecular interaction of fluquinconazole and 20-HM in the active site of 14 DM, Figure S9: Phylogenic analysis of the DNA barcoding (rcbL gene) of *G. heterophylla*, Figure S10: Phylogenic analysis of the DNA barcoding (matK gene) of *G. heterophylla* and Table S1: Summary of mass differences between theoretical and observed masses from the ESI-MS spectra.
