# Supplementary material for: In Vitro Production and Exudation of 20-Hydroxymaytenin from Gymnosporia heterophylla (Eckl. and Zeyh.) Loes. Cell Culture
Source: Plants (Basel). 2021 Jul 21;10(8):1493. doi: 10.3390/plants10081493 (PMC8398937; doi:10.3390/plants10081493)
Supplement: Supplementary file 1 [file plants-10-01493-s001.zip › plants-1228644-supplementary.pdf]

In vitro production and exudation of 20-hydroxymaytenin from *Gymnosporia heterophylla* (Eckl. and Zeyh.) Loes. cell culture

Thanet Pitakbut [1], Michael Spiteller [2] and Oliver Kayser [1,*]

[1] Technical Biochemistry, Department of Biochemical and Chemical Engineering, TU Dortmund University, 44227 Dortmund, Germany; thanet.pitakbut@tu-dortmund.de; oliver.kayser@tu-dortmund.de
[2] Institute of Environmental Research (INFU), Department of Chemistry and Chemical Biology, TU Dortmund University, 44227 Dortmund, Germany; michael.spiteller@tu-dortmund.de
* Correspondence: oliver.kayser@tu-dortmund.de

Supplementary file

Table of contents

Figure S1 HPLC chromatogram of isolated 20-HM

Figure S2 ^{1}H-NMR of 20-HM

Figure S3 ^{13}C-NMR of 20-HM

Figure S4 Proposed MS fragmentation of fragment 3 and fragment 4.1

Figure S5 Callus and cell suspension cultures of *G. heterophylla*

Figure S6 Phylogenic tree of the ITS region of the isolated *P.* cf. *olsonii* from *G. heterophylla* cell culture

Figure S7 Docking poses of fluquinconazole and 20-HM, and their average binding energy (kcal/mol)

Figure S8 2D and 3D molecular interaction of fluquinconazole and 20-HM in the active site of 14 DM

Figure S9 Phylogenic analysis of the DNA barcoding (rcbL gene) of *G. heterophylla*

Figure S10 Phylogenic analysis of the DNA barcoding (matK gene) of *G. heterophylla*

Table S1 Summary of mass differences between theoretical and observed masses from the ESI-MS spectra

Molecular docking experiment

Molecular docking was performed by following previous studies [1–3], and it was divided into three main steps. The first step was a preparation step. The second step was a setup protocol and protocol validation, and the last step was experimental. First, the crystal structure of yeast's lanosterol 14α-demethylase cytochrome P450 (14DM) complexed with fluquinconazole (PDB ID: 5EAF) was downloaded from the RCSB ProteinDataBank database (https://www.rcsb.org/ accessed on 2 June 2021) [4]. Later, water and fluquinconazole molecules were extracted from the crystal structure of 14DM using USFC chimera software (version 1.11.2) [5]. Finally, AutoDockTools (version 1.5.6) was used to prepare the extracted fluquinconazole molecule and 14DM protein structure properly for the docking experiment [6]. Second, the active site of 14DM was defined as a binding site for the experiment. This site was set as X = -10.5, y=-22.4, and z = -15.9 with a size of 18 x 18 x 18 Å. Autodock Vina (version 1.1.2) was used to perform molecular docking with default parameters [7]. After that, the setup protocol was validated by re-docking the extracted fluquinconazole molecule back to its original pose as presented in PDB ID: 5EAF. The root-mean-square deviation or RMSD value between the original and re-docked poses of fluquinconazole was evaluated. Our setup protocol provided an accurate result with a small RMSD value, less than 1 Å. Therefore, it passed an acceptance criterion reported by Shan et al. (2020) [8]. Third, the molecular docking was proceeded by using the validated setup as described earlier. Before the experiment, the chemical structure 20-HM was properly prepared as described below. In the beginning, the 2D chemical structure of 20-HM was drawn by using ACD/Chemsketch (free version, 2015), and later the 3D structure was generated after geometric and energetic (MMFF94 force field) optimization using Avogadro (version 1.2.0) [9]. This optimized 3D structure of 20-HM was then used in the docking experiment. The Viewdock package from USFC chimera and Discovery studio visualizer free version (20.1.0.19295) were used to examine docking results and generate 2D and 3D interaction diagrams for a post-docking analysis [5,10].

References

1. Phoopha, S.; Wattanapiromsakul, C.; Pitakbut, T.; Dej-Adisai, S. A New Stilbene Derivative and Isolated Compounds from *Bauhinia pottsii* Var. Pottsii with Their Anti-Alpha-Glucosidase Activity. *Pharmacogn. Mag.* **2020**, *16*, 161, doi:10.4103/pm.pm_433_19.
2. Phoopha, S.; Wattanapiromsakul, C.; Pitakbut, T.; Dej-adisai, S. Chemical Constituents of *Litsea elliptica* and Their Alpha-Glucosidase Inhibition with Molecular Docking. *Pharmacogn. Mag.* **2020**, *16*, 327, doi:10.4103/pm.pm_18_20.
3. Angarita-Rodríguez, A.; Quiroga, D.; Coy-Barrera, E. Indole-Containing Phytoalexin-Based Bioisosteres as Antifungals: In Vitro and In Silico Evaluation against *Fusarium oxysporum*. *Molecules* **2019**, *25*, doi:10.3390/molecules25010045.
4. Tyndall, J.D.A.; Sabherwal, M.; Sagatova, A.A.; Keniya, M.V.; Negroni, J.; Wilson, R.K.; Woods, M.A.; Tietjen, K.; Monk, B.C. Structural and Functional Elucidation of Yeast

Lanosterol 14α-Demethylase in Complex with Agrochemical Antifungals. *PLoS ONE* **2016**, *11*, e0167485, doi:10.1371/journal.pone.0167485.
5. Pettersen, E.F.; Goddard, T.D.; Huang, C.C.; Couch, G.S.; Greenblatt, D.M.; Meng, E.C.; Ferrin, T.E. UCSF Chimera—A Visualization System for Exploratory Research and Analysis. *J. Comput. Chem.* **2004**, *25*, 1605–1612, doi:10.1002/jcc.20084.
6. Morris, G.M.; Huey, R.; Lindstrom, W.; Sanner, M.F.; Belew, R.K.; Goodsell, D.S.; Olson, A.J. AutoDock4 and AutoDockTools4: Automated Docking with Selective Receptor Flexibility. *J. Comput. Chem.* **2009**, *30*, 2785–2791, doi:10.1002/jcc.21256.
7. Trott, O.; Olson, A.J. AutoDock Vina: Improving the Speed and Accuracy of Docking with a New Scoring Function, Efficient Optimization, and Multithreading. *J. Comput. Chem.* **2010**, *31*, 455–461, doi:10.1002/jcc.21334.
8. Shah, Z.A.; Abu-Izneid, T.; Rauf, A.; Rashid, U.; Nizam, M.; Muhammad, N.; Rengasamy, K.R.R. Phosphodiesterase 1 Inhibition and Molecular Docking Study of Phytochemicals Isolated from Stem Heartwood of *Heterophragma adenophyllum* Seem. *S. Afr. J. Bot.* **2020**, *135*, 274–279, doi:10.1016/j.sajb.2020.08.013.
9. Hanwell, M.D.; Curtis, D.E.; Lonie, D.C.; Vandermeersch, T.; Zurek, E.; Hutchison, G.R. Avogadro: An Advanced Semantic Chemical Editor, Visualization, and Analysis Platform. *J. Cheminformatics.* **2012**, *4*, 17, doi:10.1186/1758-2946-4-17.
10. Biovia, D.S. Discovery Studio Visualizer. *San Diego, CA, USA* **2017**, *936*.

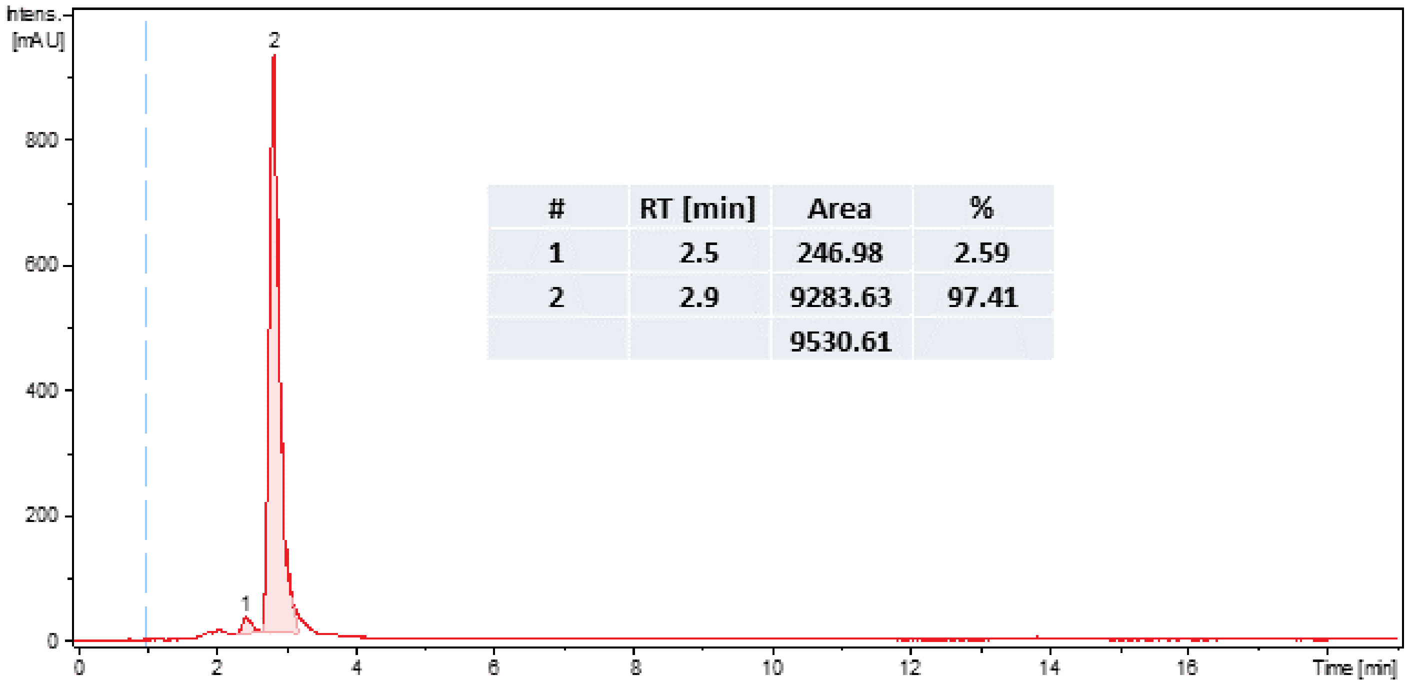

#	RT [min]	Area	%
1	2.5	246.98	2.59
2	2.9	9283.63	97.41
		9530.61	

Figure S1 HPLC chromatogram of isolated 20-HM.

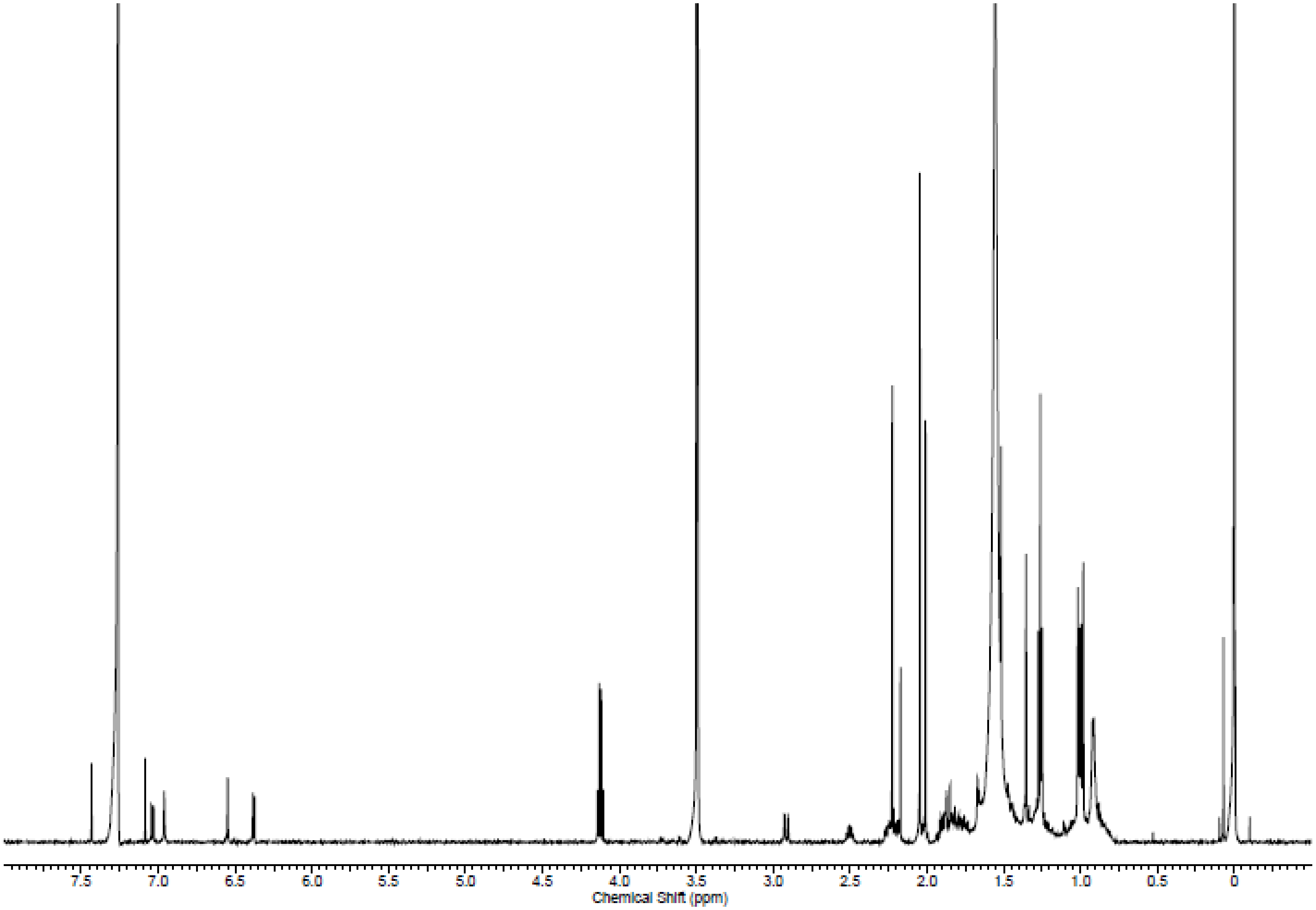

Figure S2 ^{1}H-NMR of 20-HM.

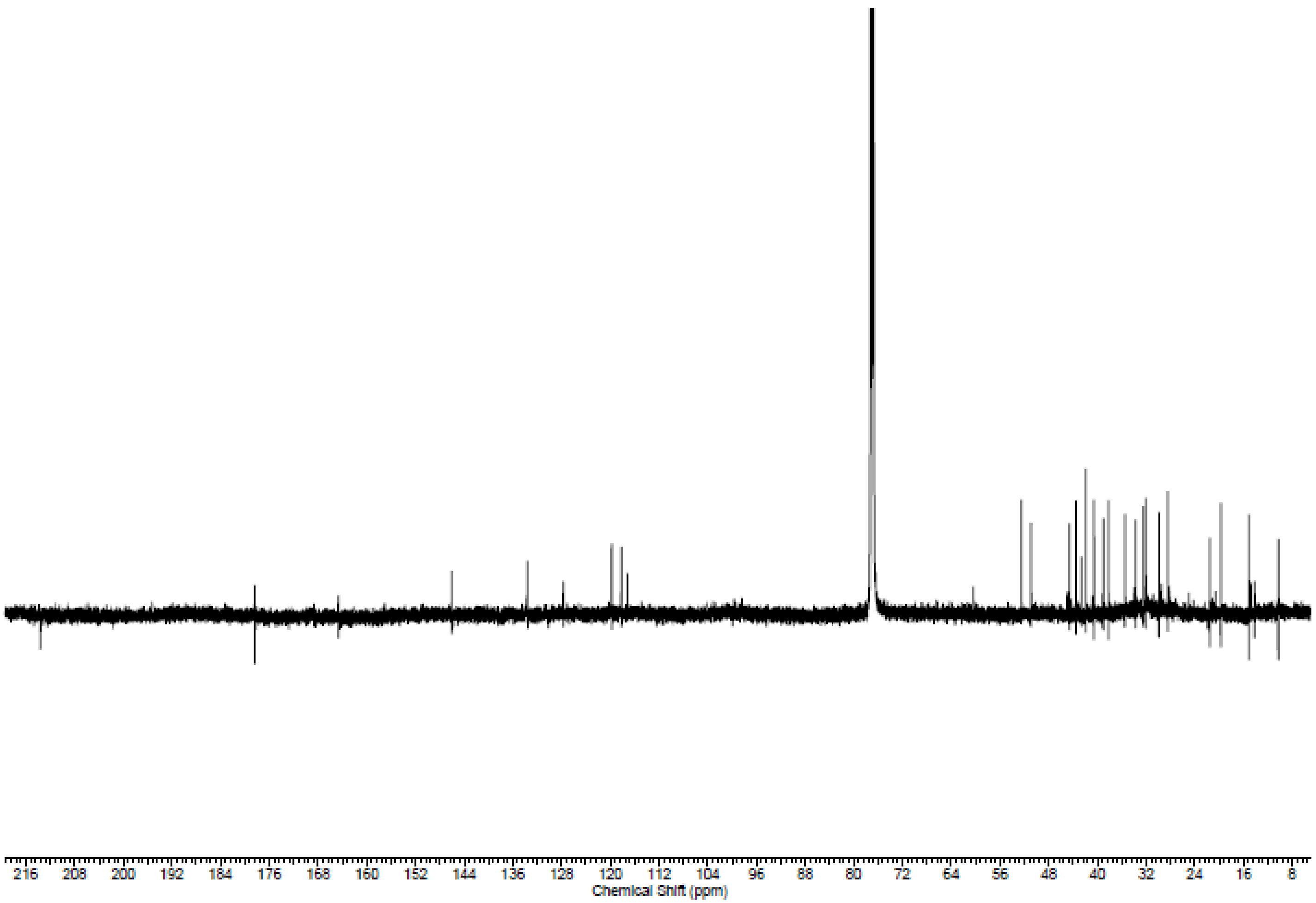

Figure S3 ^{13}C-NMR of 20-HM.

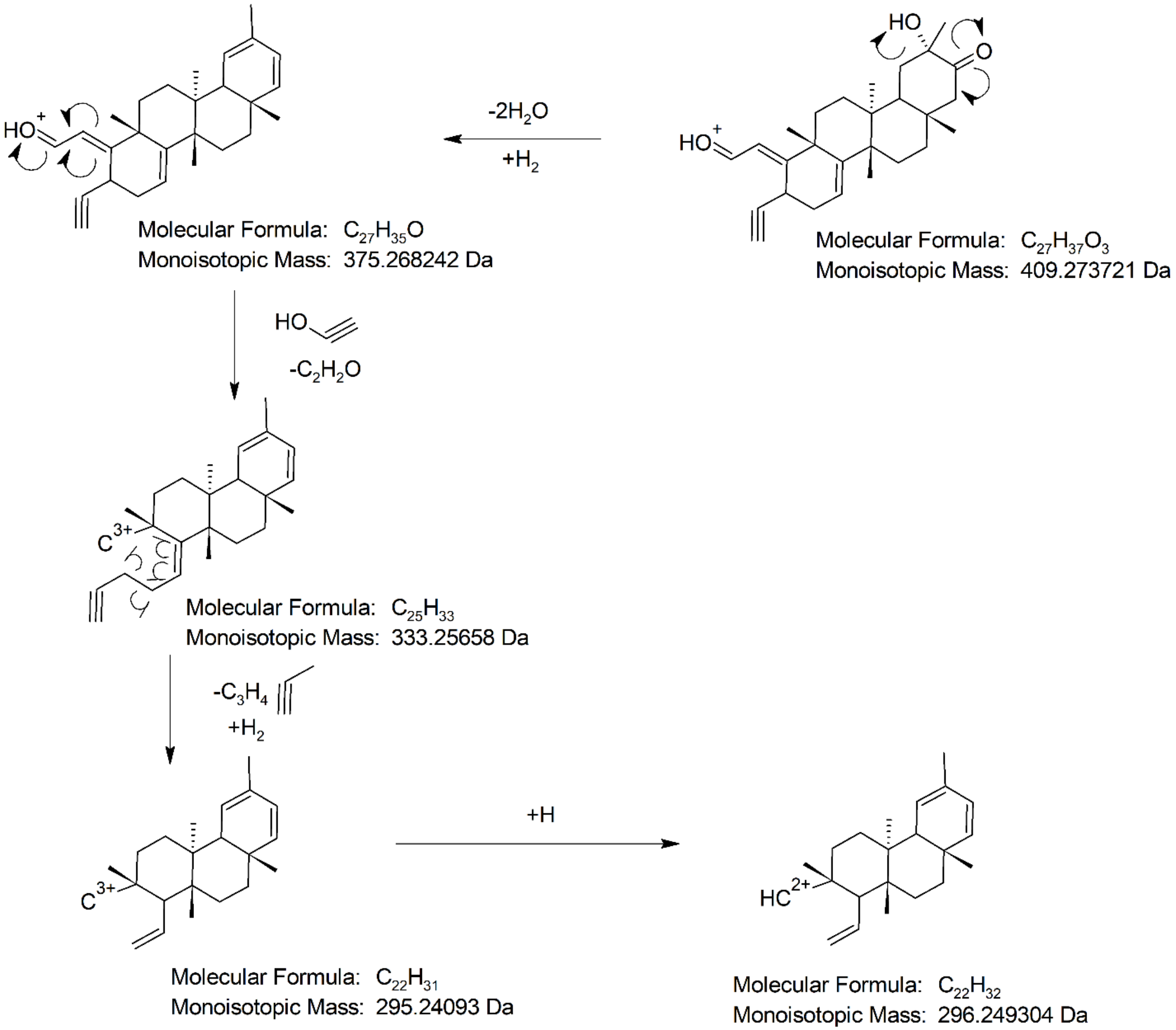

Figure S4 Proposed MS fragmentation of fragment 3 and fragment 4.1.

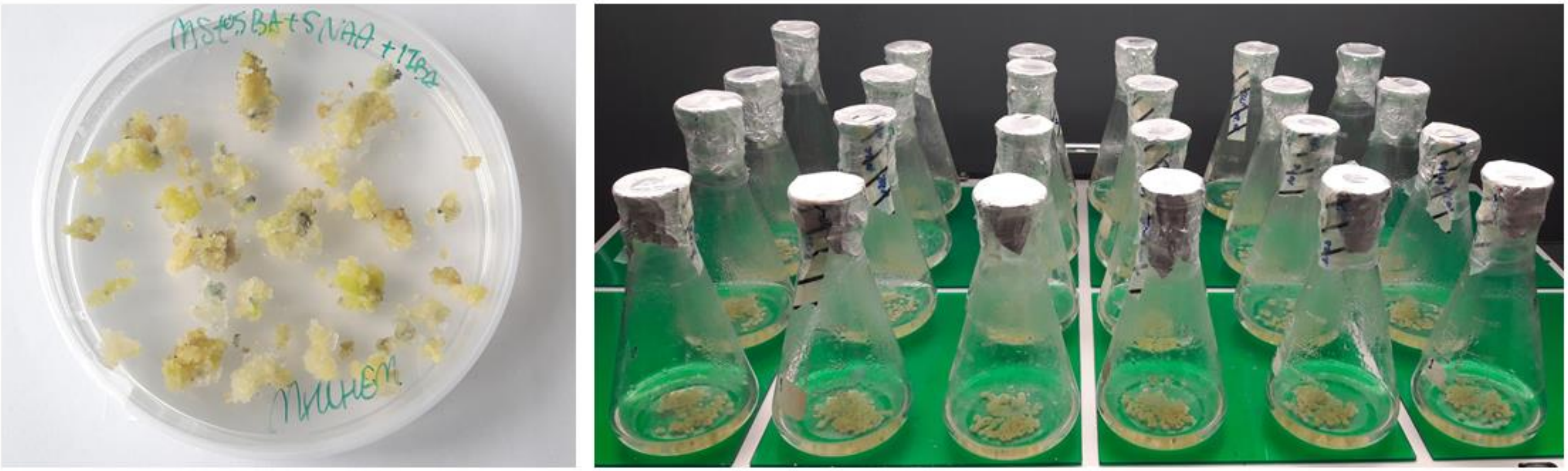

Figure S5 Callus (left) and cell suspension (right) cultures of *G. heterophylla.*

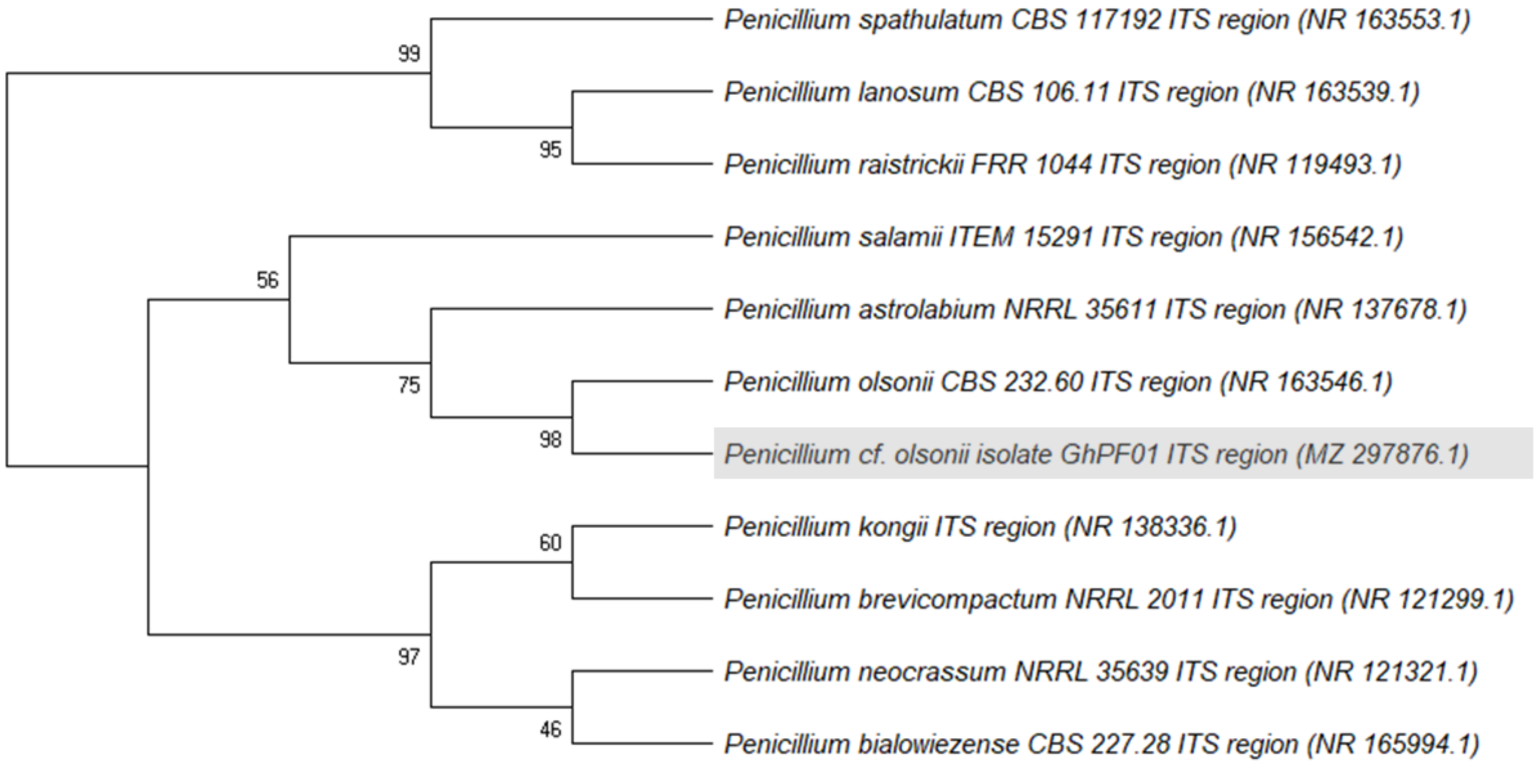

Figure S6 Phylogenic tree of the ITS region of the isolated *P.* cf. *olsonii* from *G. heterophylla* cell culture and related species according to the Blastn analysis (selecting ITS database for fungi type and reference materials) (TreeBASE tree ID: tr131462). Multiple alignments are performed using the Muscle tool, and the phylogenic tree is constructed using the Maximum Likelihood method based on the Tamura-Nei model from MEGA-X software (Version 10.0.4). The bootstrap values are shown on the branch based on 1,000 pseudoreplicates. Initial tree(s) for the heuristic search is received by using Neighbor-Join and BioNJ algorithms. The grey box indicates the isolated *P.* cf. *olsonii* from *G. heterophylla* cell culture.

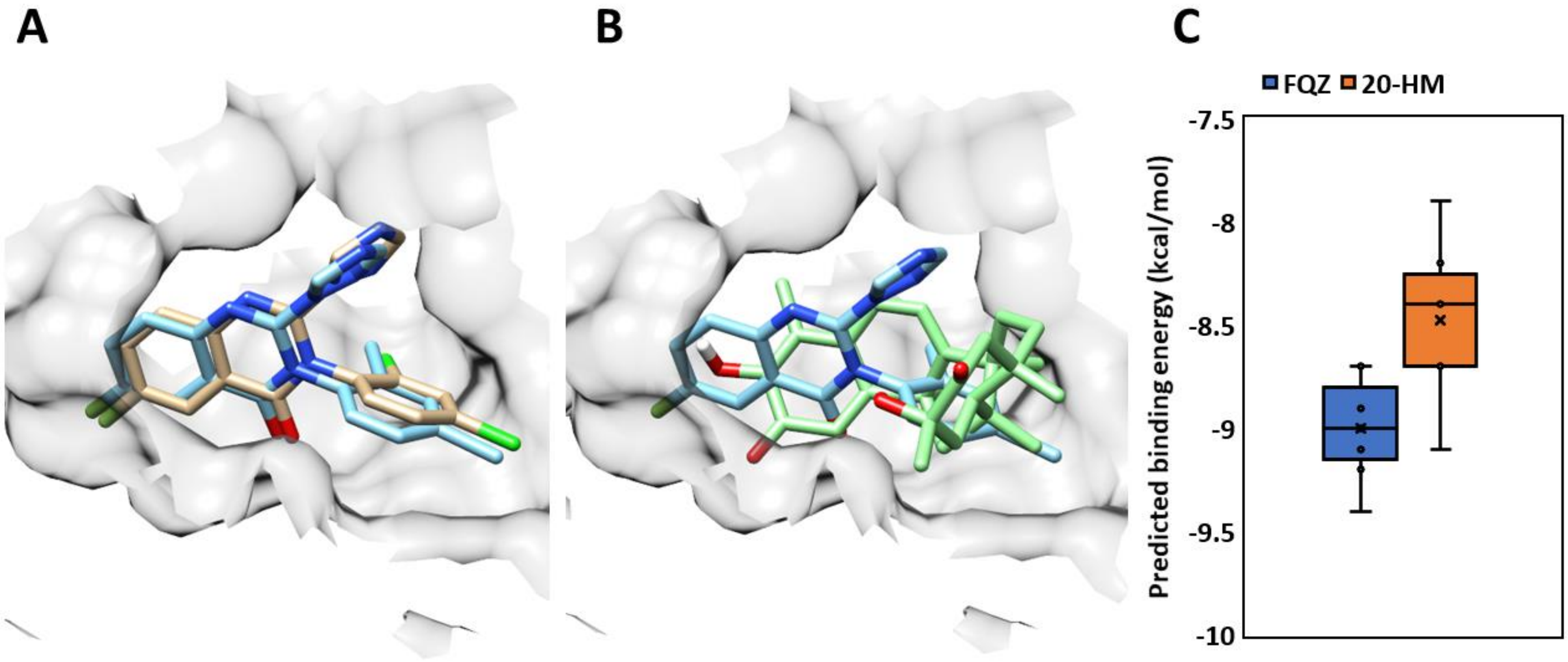

Figure S7. Structural alignments of the original and re-docking poses of fluquinconazole (FQZ) present in brown and blue colors accordingly (A); and superimposition of docking FQZ and 20-HM presents in blue and green colors, respectively (B); and average binding energy values (kcal/mol) of FQZ and 20-HM are presented in a box and whisker plot (C). USFC Chimera (version 1.11.2) is used to visualize all 3D structures.

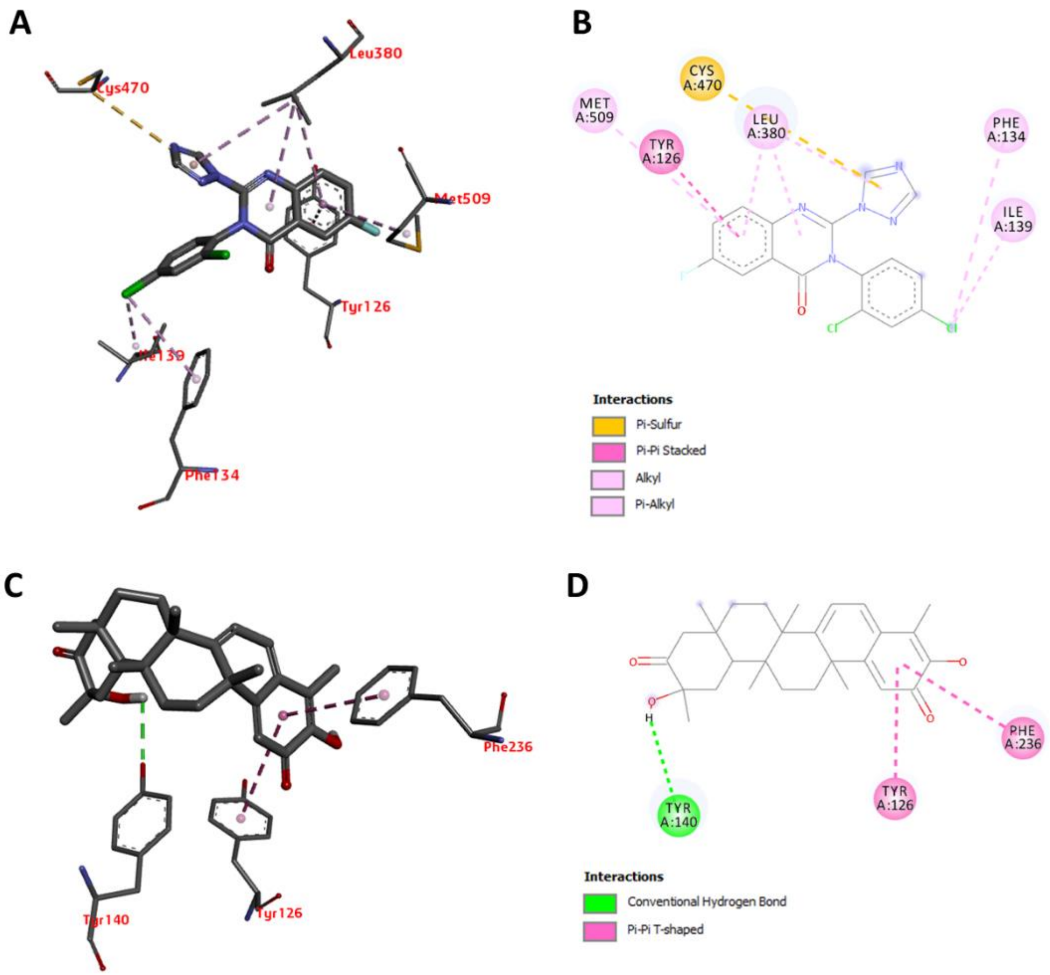

Figure S8. 3D and 2D interaction diagrams between fluquinconazole and amino acids in the active site of yeast's 14MD present in (A) and (B); where 3D and 2D interaction diagrams between 20-HM and amino acids are presented in (C) and (D). Discovery studio visualizer free version (20.1.0.19295) is used to obtain 2D and 3D diagrams.

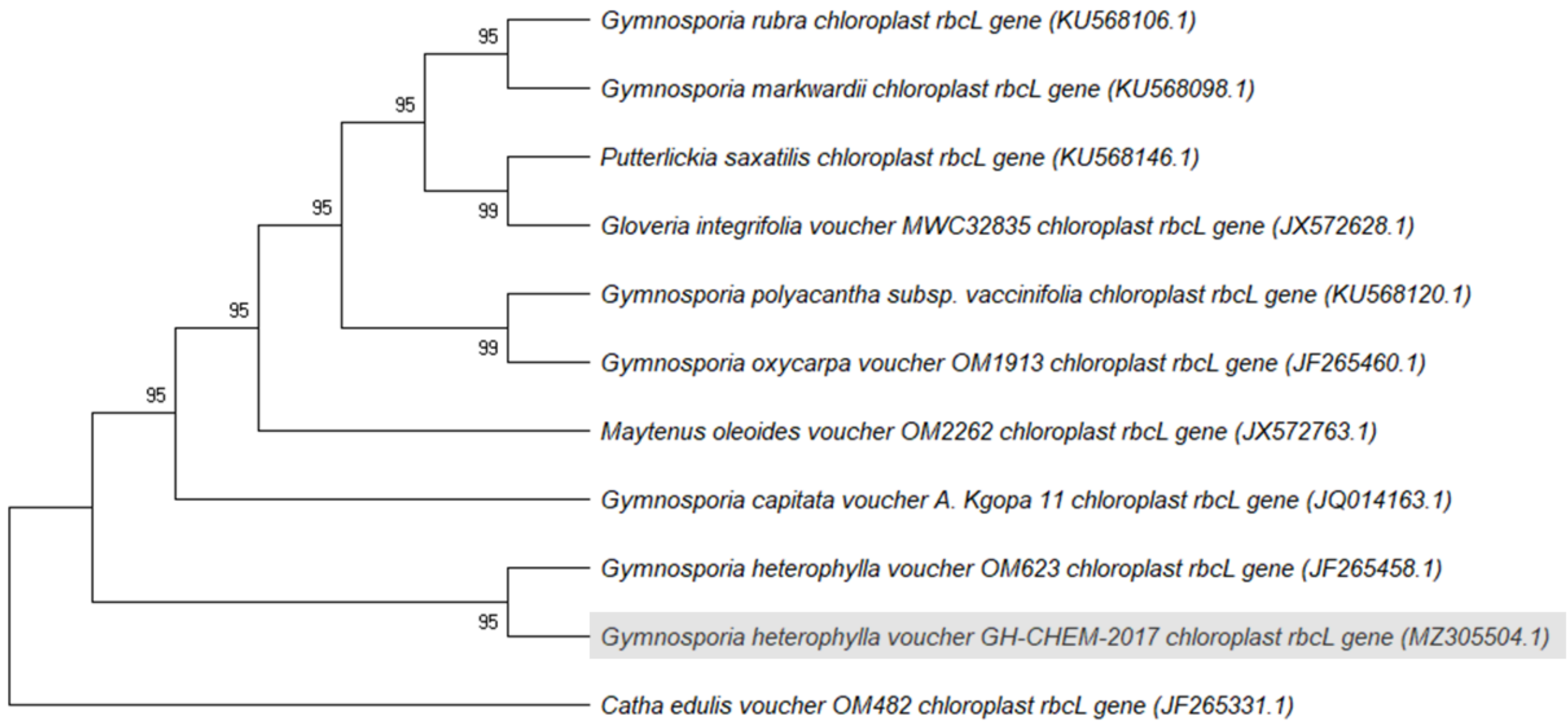

Figure S9. Phylogenic tree of the rcbL gene of our *G. heterophylla* sample and the related species according to the Blastn analysis (TreeBASE tree ID: tr131454). Multiple alignments are performed using the Muscle tool, and the phylogenic tree is constructed using the Maximum Parsimony (MP) method from MEGA-X software (Version 10.0.4). The bootstrap values are shown on the branch based on 1,000 pseudoreplicates. The MP tree is using the Subtree-Pruning-Regrafting (SPR) algorithm with search level 1. The grey box indicates the rcbL gene from our *G. heterophylla* sample.

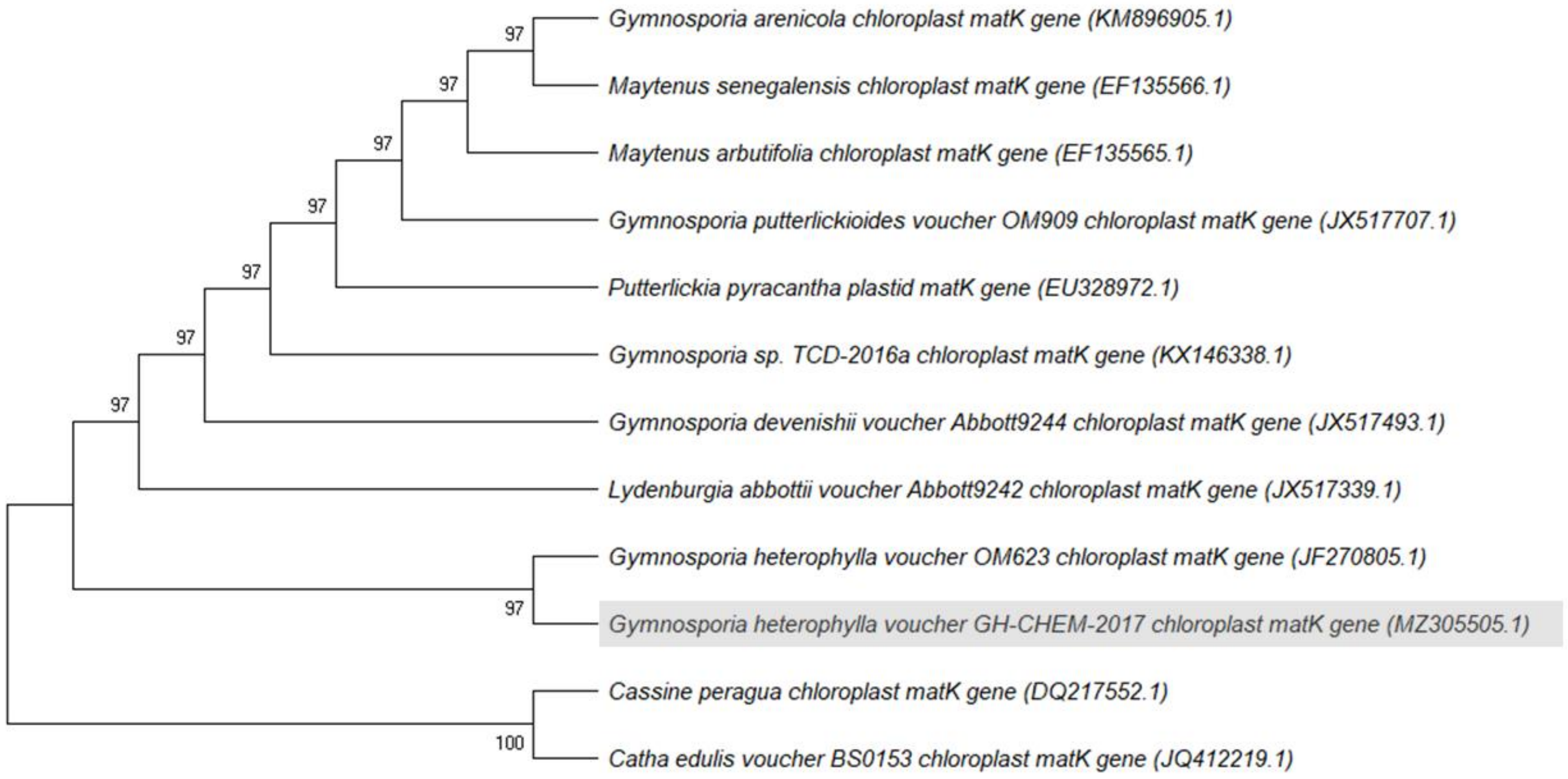

Figure S10. Phylogenic tree of the matK gene of our *G. heterophylla* sample and the related species according to the Blastn analysis (TreeBASE tree ID: tr131441). Multiple alignments are performed by using the Muscle tool, and the phylogenic tree is constructed by the Maximum Parsimony (MP) method from MEGA-X software (Version 10.0.4). The bootstrap values are shown on the branch based on 1,000 pseudoreplicates. The MP tree is using the Subtree-Pruning-Regrafting (SPR) algorithm with search level 1. The grey box indicates the matK gene from our *G. heterophylla* sample.

TableS1 Summary of mass differences between theoretical and observed masses from the ESI-MS spectra.

Name	Observed Mass (Da)	Predicted Mass (Da)	Difference Mass		Chemical Formula	± 5 mDa	± 10 Da
			Da	mDa			
[Moleucular ion peak]$^{+}$	437.2626	437.2686	-0.0060	-6	C28H37O4	No	Yes
[Fragment 1]$^{+}$	421.2721	421.2737	-0.0016	-1.6	C28H37O3	Yes	Yes
[Fragment 2]$^{+}$	419.2545	419.2581	-0.0036	-3.6	C28H35O3	Yes	Yes
[Fragment 3]$^{+}$	409.2723	409.2737	-0.0014	-1.4	C27H37O3	Yes	Yes
[Fragment 4.1]$^{+}$	296.2580	296.2493	0.0087	8.7	C22H32	No	Yes
[Fragment 4.2]$^{+}$	296.2580	296.2584	-0.0004	-0.4	C18H34NO2	Yes	Yes
[Fragment 4.1-H+Na]$^{+}$	318.2395	318.2317	0.0078	7.8	C22H31Na	No	Yes
[Fragment 4.1-H+H2O+Na]$^{+}$	336.2500	336.2423	0.0077	7.7	C22H33ONa	No	Yes
[Fragment 5]$^{+}$	280.2617	280.2634	-0.0017	-1.7	C18H34NO	Yes	Yes
[Base peak]$^{+}$	201.0902	201.0910	-0.0008	-0.8	C13H13O2	Yes	Yes
